# Challenging biological limits with microfluidic single cell analysis

**DOI:** 10.1111/1751-7915.12252

**Published:** 2015-01-28

**Authors:** Christian Dusny, Andreas Schmid

**Affiliations:** Department Solar Materials, Helmholtz Centre for Environmental Research – UFZ04318, Leipzig, Germany

Back in the 17th century, van Leeuwenhoek was the first to advance into the microbial universe with his simple, but powerful single lens microscope. In a drop of aqueous infusion, he saw for the first time the basic functional and replicating units of life: single cells, in the shape of individual microbes. This observation laid the foundation for modern (micro-) biology. What began with a single cell was followed by centuries of research expanding our knowledge about microbial functionality and cellular processes – with population-based experiments. The central paradigm of microbiological methodology was reductionism, comprising isolation of microorganisms from their natural ecosystems to study them in axenic cultures in artificial environments like shake flasks or bioreactors. This determination of the boundaries of biological systems allowed controlling genetic identity, macroscopic cultivation parameters like availability and type of carbon source, as well as physicochemical parameters. Interactions of microbes with their environment in terms of mass and energy exchanges could be studied by microbial population ecology. Conclusions were and are drawn for idealized hypothetical single cells, de facto serving as wildcards in many areas from population function, to physiology or molecular biology. Yet, cellular individuality in isogenic populations is a fact.

To what extent can the knowledge obtained from homogenized results of population experiments be really transferred to individual cells? Strictly speaking, it is not possible to decouple intracellular stochastic processes like molecule location and abundance, and external contributions to cellular individuality due to the lack of environmental control in classical shake flask or bioreactor experiments, including continuous chemostats. Only advances in microstructure technology and its symbiosis with microbiology during the past two decades made it technically and conceptually feasible to tackle these important biological questions. New microfluidic single cell isolation, analysis and cultivation methods, matching the scale of cultivation space and the dimensions of single microorganisms, introduced the possibility to control tiny amounts of liquid volume and manipulate the extracellular environment for defined physicochemical perturbations during the cultivation of single cells. Up to now, this basic concept of microfluidic single cell analysis represents the ultimate increment of the microbiological reductionist paradigm, decoupling environment and dynamics of cellular processes in a controlled single cell microecosystem (Kortmann *et al*., 2009).

We are now witnessing increasing scientific interest and applications of microfluidic single cell analysis. It is becoming a standard technology in many microbiology and biotechnology laboratories, not at least due to the facts that several commercial microcultivation and analysis platforms are already available and established technologies like polydimethylsiloxane (PDMS) molding allow the design and fabrication of custom microfluidic networks, from prototype to final devices in short time periods. Single cells and mixed populations can be spatially and temporally organized in defined habitats. The possibility to control physicochemical cues and interactions between individual microbes in technical or natural ecosystems might well set off a revolution in microbial ecology. Microfluidic single cell assay formats already resulted in staggering insights into the cell as the basic functional biological unit. Examples, to name but a few, cover studies of mutual auxotrophy compensation in mixed, spatially adjacent cultures (Moffitt *et al*., 2012), ageing in single yeast (Lee *et al*., 2012) and single bacteria (Wang *et al*., 2010), interrelation of microbial growth rate and extracellular environment (Dusny *et al*., 2012), growth and gene expression (Sweedler and Arriaga, 2007) single cell dynamics during nutrient shifts (Boulineau *et al*., 2013) and effects of spatial confinement, like growth in narrow pores, on reproductive traits (Mannik *et al*., 2012). Studies like these demonstrate that microfluidic analyses of single cell dynamics, which began as a tender liaison between microengineering and microbiology, develop into basic pillars of systems and synthetic biology. Moreover, microfluidic single cell technologies might soon open to microbiology what was previously reserved to mammalian single cells: simultaneous genome and transcript sequencing (Blainey, 2013) of isolated single cells, even under transient conditions. The technologies are rapidly developing and already enable the mapping of metagenomes in complex microbial consortia.

The potential of microfluidic single cell analysis is vast. New microbioreactor concepts and massively improved analytical technologies will allow the formulation and testing of new hypotheses. The main driver for future progress in single cell analysis will not only be the development of new or improved single cell technologies. It will again be the creativity and demand of microbiologists and biotechnologists for specialized (or universal) tools to answer specific biological questions and to understand the functional aspects of a single cell. Conceivable applications for new single cell technologies are manifold. One target is the long discussed cellular designer chassis as a highly specialized microbial factory for understanding functionality but also for engineering specific biotechnological purposes. The rational development of such cell factories assumes that control circuits and complex interconnections and relationships in the intracellular hierarchy, from genome to metabolism and their regulation in response to environmental stimuli, are known. A final systems-level understanding of cellular processes for predicting metabolic activity can only be obtained via controlled single cell experiments comprising all cellular responses to standardized perturbations, unbiased from population activity. Computational modelling of cells as systems and the assignment of catalytic and regulatory function to cellular individuality, and finally the design of custom cells for implementation into specific biotechnological processes will come within reach. We will also be in a position to experimentally address population dynamics and function approaching from the microbial individual.

It is also clear that this access to microbial individuality will diversify and differentiate to satisfy future demands of various scientific fields. It might help our understanding of the interplay between the extracellular environment and cellular processes in natural ecosystems, mixed cultures and isogenic populations in technical ecosystems (reactors), as used in environmental applications, medical research and industrial processes. It will also be of utmost importance to adapt basic microbial methods to the analysis of single cell parameters in a classical sense. This applies in particular to a standardized quantification of cellular parameters like rate of biogenesis, maintenance, substrate uptake, as well as product and metabolite efflux. Even most fundamental cellular characteristics, like the specific growth rate of a cell, typically described by biomass or cell volume increase, are inaccessible at the level of a single cell because a unified methodology is lacking. These parameters are the keys for the systematic description of single cells. They will finally identify the individual contribution of single cells to the macroscopic output of microbial populations in processes in natural and technical ecosystems.

Reductionism in the area of single cell analysis might even go further. In contrast to top-down approaches, comprising *in vivo* studies of single cells as whole functional systems, biomimetic bottom-up research based on *in vitro* assays with liposomes and vesicles as artificial cell-like systems are already emerging and will enrich ‘classical’ life cell approaches (Ullman *et al*., 2007). Mimicking a biological cell with the cell membrane maintaining a non-equilibrium state of the system with its environment, the analysis of specific features like transmembrane transport or the measurement of enzyme kinetics under *in vivo*-like reaction conditions will become possible. This will greatly enhance our understanding of processes in a single cell – and in life in general. However, even for this ultimate stage of reductionism, the description of artificial cell-like systems still requires classical (micro-) biological methodology. Microfluidic single cell approaches, in tandem with a holistic understanding of cellular traits at the level of a single cell, will lead to exciting discoveries that deepen our understanding of the single cell as the elementary unit of life.

## Conflict of interest

None declared.
